# Durvalumab as Consolidation Therapy in Post-Concurrent Chemoradiation (CCRT) in Unresectable Stage III Non-Small Cell Lung Cancer Patients: A Multicenter Observational Study

**DOI:** 10.3390/vaccines9101122

**Published:** 2021-10-01

**Authors:** Chin-Chou Wang, Li-Chung Chiu, Jia-Shiuan Ju, Yu-Ching Lin, Yueh-Fu Fang, Cheng-Ta Yang, Ping-Chih Hsu

**Affiliations:** 1 Division of Pulmonary & Critical Care Medicine, Kaohsiung Chang Gung Memorial Hospital, Kaohsiung City 83301, Taiwan; ccwang5202@yahoo.com.tw; 2 Department of Medicine, College of Medicine, Chang Gung University, Taoyuan City 33302, Taiwan; pomd54@cgmh.org.tw (L.-C.C.); lin0927@cgmh.org.tw (Y.-C.L.); 3 Division of Thoracic Medicine, Department of Internal Medicine, Chang Gung Memorial Hospital at Linkou, Taoyuan City 33305, Taiwan; b9502008@cgmh.org.tw (J.-S.J.); dr.fang.yf@gmail.com (Y.-F.F.); Yang1946@cgmh.org.tw (C.-T.Y.); 4 Department of Thoracic Medicine, New Taipei Municipal Tu Cheng Hospital, New Taipei City 23652, Taiwan; 5 Division of Thoracic Oncology, Department of Respiratory and Critical Care Medicine, Chang Gung Memorial Hospital, Chiayi Branch, Chiayi City 61363, Taiwan; 6 Department of Respiratory Care, Chiayi Campus, Chang Gung University of Science and Technology, Chiayi City 33303, Taiwan; 7 Department of Internal Medicine, Taoyuan Chang Gung Memorial Hospital, Taoyuan City 33378, Taiwan; 8 Department of Respiratory Therapy, College of Medicine, Chang Gung University, Taoyuan City 33302, Taiwan

**Keywords:** durvalumab, stage III non-small cell lung cancer (NSCLC), concurrent chemoradiation (CCRT), programmed death-ligand 1 (PD-L1), immune checkpoint inhibitors (ICIs), epidermal growth factor receptor (EGFR) mutation

## Abstract

Background: The experience of using consolidation durvalumab in post-concurrent chemoradiation (CCRT) unresectable stage III non-small cell lung cancer (NSCLC) is rare in real-world clinical practice, and the factors associated with its efficacy are also unclear. We sought to analyze the efficacy of consolidation durvalumab and the factors associated with its efficacy using a multicenter observational study. Methods: The data for 61 patients with post-CCR unresectable stage III NSCLC receiving consolidation durvalumab at the Chang Gung Memorial Hospitals in Linkou, Keelung, Chiayi, and Kaohsiung from November 2017 to March 2020 were analyzed. (3) Results: The median post-CCRT progression-free survival (PFS) and time to metastatic disease or death (TMDD) for consolidation durvalumab were 14.0 months and 16.7 months, respectively. In multiple variant factors analysis, we found that an epidermal growth factor receptor (EGFR) mutation was an independently unfavorable predictive factor for consolidation durvalumab therapy regarding PFS. The median post-CCRT PFS was 6.50 months for EGFR-mutated patients and 33.63 months for EGFR wild-type and unknown patients (HR = 10.47; 95% CI, 4.55–24.07; *p* < 0.001). Conclusions: Consolidation durvalumab is effective and safe for post-CCRT unresectable stage III NSCLC in clinical practice, but EGFR mutation is an unfavorable factor for consolidation durvalumab. Thus, searching for a better consolidation therapy for EGFR-mutated patients is warranted.

## 1. Introduction

Stage III non-small cell lung cancer (NSCLC) accounts for 30% of all stages of NSCLC at initial diagnosis [1,2]. The presentation of stage III NSCLC is very heterogeneous and appears from locally bulky tumors to small lung tumors with multiple mediastinal lymph node metastases [3,4]. The management of a stage III disease is complex, and a single treatment modality alone—including surgery, radiation therapy (RT), or chemotherapy—is not adequate. Therefore, multimodality therapy is suggested for stage III NSCLC patients with an adequate cardio-pulmonary function reserve and good performance status [3,4,5]. Currently, neoadjuvant concurrent chemoradiation therapy (CCRT) is the preferred treatment strategy for stage III NSCLC, because CCRT has been reported to achieve 50%–70% objective response rates in previous studies. Chemotherapy can limit the cell cycle in the G2/M phase and can increase the cytotoxicity of radiation to cancer cells [6,7]. This explains why CCRT has higher response rates than sequential chemoradiation therapy [6,7]. Tumor resection surgery is feasible for some stage III NSCLC patients whose disease is controlled by neoadjuvant CCRT. Previous studies have shown that stage III patients receiving complete resection after neoadjuvant CCRT experience better survival outcomes than those receiving definite CCRT only [8,9]. Unfortunately, most stage III NSCLC patients (>50%) still have an unresectable disease, even after neoadjuvant CCRT [4,8,9]. Consolidation chemotherapy following neoadjuvant CCRT is administrated for some unresectable patients, but the survival benefit of consolidation is very limited according to the reports of previous studies [10,11].

Immunotherapy targeting the programmed cell death protein-1 (PD-1)/programmed death-ligand 1 (PD-L1) checkpoint has been developed and is widely used in cancer therapy, including for NSCLC [12,13]. Three anti-PD-1/PD-L1 immune checkpoint inhibitors (ICIs) (i.e., pembrolizumab, nivolumab, and atezolizumab) have been approved for the treatment of metastatic NSCLC based on several large and pivotal clinical trials [13,14,15]. In previous large pivotal clinical trials, anti-PD-1/PD-L1 ICIs with or without chemotherapy significantly improved the survival rates of metastatic NSCLC patients when compared with conventional chemotherapy alone [13,14,15]. Durvalumab (an anti-PD-L1 ICI) used as a consolidation therapy in post-CRT unresectable stage III NSCLC was explored in the PACIFIC trial. Durvalumab was shown to significantly improve the progression-free survival (PFS), two-year survival rate, and overall survival (OS) when compared with a placebo in the PACIFIC study [16].

Previous preclinical and clinical studies have reported that chemotherapy and radiation therapy enhance the anti-tumor effect of anti-PD-L1 antibodies [17,18,19,20]. In the tumor immune microenvironment, immunogenic cell death (ICD) is involved in the adaptive stress response to facilitate the maturation of dendritic cells (DCs). Myeloid-derived suppressor cells (MDSCs) and T (Treg) cells in the tumor microenvironment downregulate anti-tumor immunity and promote tumor progression. Conventional chemotherapy regulates the immunogenicity of tumor cells by promoting ICD and by suppressing MDSCs and Treg cells [17,18,19]. A previous preclinical study demonstrated that chemotherapy increased the anti-tumor effect of anti-PD-1 antibodies by promoting the ICD pathway in a mouse model [17]. Local radiation therapy damages tumors and induces the release of neoantigens and damage-associated molecular patterns (DAMPs) from tumor cells. Tumor neoantigens (also called tumor-associated antigens (TAAs)) prime T cell-mediated anti-tumor immunity (abscopal effect) when traveling to lymph nodes. DAMPs destroy tumor-supporting stroma, which maintain the immunosuppressive effect of tumor cells [18,20]. Taken together, these factors explain how the addition of chemotherapy and/or radiation therapy to anti-PD-L1 ICIs improves the survival of NSCLC patients in clinical studies [14,21]. Based on above theory, the PACIFIC trial was conducted and demonstrated a promising effect for durvalumab in improving the survival of post-CCRT stage III unresectable NSCLC patients [16].

Although the PACIFIC trial demonstrated a promising efficacy of durvalumab in post-CCRT unresectable stage III NSCLC, real-world clinical experience using durvalumab as a consolidation therapy is very rare. Herein, we conducted a multicenter observational study to investigate the efficacy of durvalumab as a consolidation therapy following CCRT in unresectable stage III NSCLC in clinical practice and analyzed the clinical factors affecting the efficacy of durvalumab.

## 2. Materials and Methods

### 2.1. Patients

**Figure 1:**
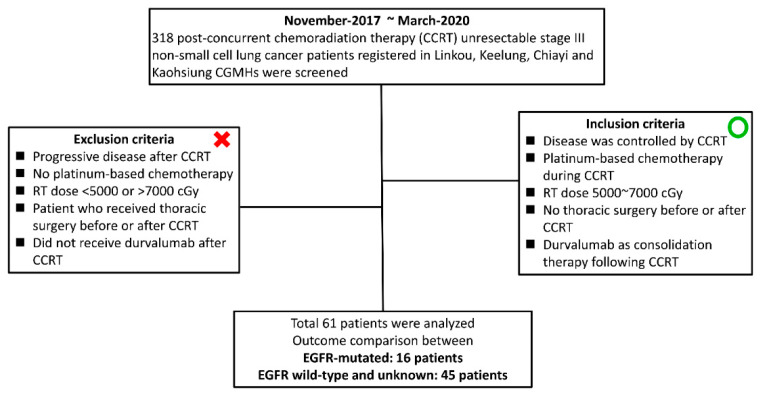
Flowchart of patient recruitment in this study.

Between November 2017 and March 2020, 318 post-concurrent chemoradiation therapy (CRT) unresectable stage III histologically diagnosed NSCLC patients registered in the cancer centers of four medical institutions (the Linkou, Keelung, Chiayi, and Kaohsiung Chang Gung Memorial Hospitals) were screened. The inclusion criteria were as follows: (1) disease controlled by CRT; (2) platinum-based chemotherapy administered during CRT; (3) a radiation therapy (RT) dose between 5000 and 7000 centigray (cGy); (4) no history of receiving thoracic surgery before or after CRT; and (5) durvalumab as a consolidation therapy following definite CRT. The exclusion criteria were as follows: (1) progressive disease after definite CRT; (2) patients not receiving platinum-based chemotherapy regimens during CRT; (3) an RT dose less than 5000 cGy or higher than 7000 cGy; (4) receiving thoracic surgery either before or after CRT; and (5) not receiving durvalumab after CRT. Sixty-one patients were finally retrieved and analyzed. A flow diagram with the inclusion and exclusion criteria for recruiting patients in this study is shown in Figure 1.

The patients’ clinical medical records, treatment information, and treatment-related adverse effects (AEs) were retrospectively retrieved from electronic medical records. According to the protocol of Chang Gung Medical Foundation Cancer Center, each patient received computed tomography (CT), fluorodeoxyglucose (FDG)-positron emission tomography (PET) scans, and brain magnetic resonance imaging (MRI) at diagnosis to determine the stages. All patients regularly had whole-body CT examinations to follow the disease status during treatment. Patients received additional images, including chest plain films, sonograms, FDG-PET scans, and MRIs during treatment and follow-up based on the clinical physicians’ judgments and needs. The last time point of follow-up in this study was March 2021.

Other molecular and biomarker tests, including epidermal growth factor receptor (EGFR) mutations and tumor PD-L1 expression, were performed at the request of the physicians for the treatment plan. The EGFR mutations were assayed using an amplified refractory mutation system, i.e., Scorpion (ARMS/S), or next-generation sequencing (NGS). The tumor surface PD-L1 expression was stained using an immunohistochemistry (IHC) 22C3 pharmDx assay (Dako North America) [22].

### 2.2. Evaluation of the CRT Response, Survival, and Treatment-Induced AEs of Durvalumab

The treatment response of CRT was evaluated by Response Evaluation Criteria in Solid Tumors (RECIST) version 1.1 and defined as partial response (PR) or stable disease (SD). The PFS was defined as the time from the first date of administrating durvalumab until the date of the first PD images or last visit. Time to metastatic disease or death (TMDD) was defined as the duration from the first date of durvalumab dose administration until the date of the first metastasis image or death. OS was defined from the date of diagnosis until the date of death. If patients were alive at the last follow-up (31 March 2021), survival was noted for the last visit date recorded.

Durvalumab-related AEs were retrieved from the medical records in follow-up visits. AEs were graded according to the National Cancer Institute Common Terminology Criteria for Adverse Events, version 4.0.

### 2.3. Statistical Analysis

The baseline demographic characteristics and treatment information of the patients in our study are presented as quantitative variables. The survival analysis, including PFS, TMDD, and OS, were estimated using Kaplan–Meier curves. The comparison of PFS between different variables was analyzed using Cox regression with univariate analysis as well as multivariate analysis. The Mann–Whitney test was used to analyze the statistical significance of continuous variables between two groups. The Fisher’s exact and Chi-square tests were used for categorical variables. The cutoff value of the neutrophil-to-lymphocyte ratio (NLR) was estimated using the area under the curve (AUC). All *p*-values were two-sided, and a *p*-value < 0.05 was defined as statistically significant. All of the data of this study were analyzed using IBM SPSS Statistics version 22.0 (SPSS Corp., Chicago, IL, USA). The figures of all survival curves were plotted using GraphPad Prism (Version 5.0; GraphPad Software, San Diego, CA, USA).

## 3. Results

### 3.1. Baseline Demographic Characteristics and CRT Treatment Information of the Study Patients

The baseline demographic characteristics and CRT treatment information of the 61 patients who received post-CRT durvalumab consolidation therapy are summarized in Table 1. Forty-nine patients in this study received genomic tests for EGFR and anaplastic lymphoma kinase (ALK) mutations: 16 were EGFR-mutated, 33 had wild-type EGFR, and none had an ALK mutation. Among the 16 patients with an EGFR mutation, 9 were L858R mutations, 6 were exon 19 deletion mutations, and 1 was a G719X mutation. Regarding the histological type among the 49 patients receiving EGFR mutation tests, 42 were adenocarcinomas, 5 were squamous cell carcinomas, and 2 were not-otherwise-specified NSCLCs. In the 16 EGFR-mutated patients, 15 had adenocarcinomas and 1 had a not-otherwise-specified NSCLC. For the treatment response to neoadjuvant CCRT, 34 patients achieved PR and 27 patients had SD. The median time from the end of CCRT to the first time point of durvalumab administration was 1.8 months. The median follow-up time in this study was 27.0 months.

### 3.2. Efficacy of Durvalumab and the Predictive Factors Associated with PFS

**Figure 2:**
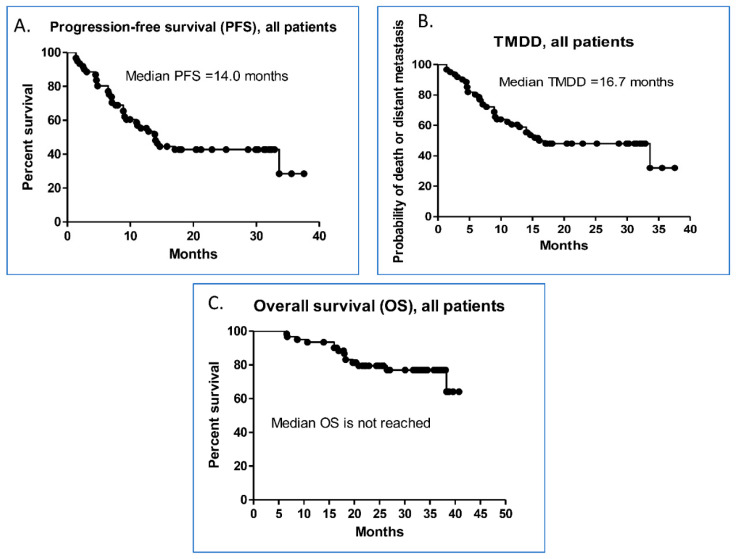
Kaplan–Meier survival curves of the (**A**) PFS, (**B**) TMDD, and (**C**) OS of all study patients.

For all 61 patients, the median PFS was 14.0 months (95% confidence interval (CI), 10.42–17.58; Figure 2A) and the median TMDD was 16.7 months (95% CI, 5.36–26.78; Figure 2B). The median OS was not reached through the last follow-up data in this study (Figure 2C). The median PFS with different predictive variables was analyzed (Table 2). In the univariate analysis, the factor significantly associated with shorter PFS was an EGFR mutation (6.5 vs. 33.63 months, *p* < 0.001). Adenocarcinoma demonstrated a shorter PFS trend, which did not reach statistical significance (*p* = 0.075). The Cox regression model identified that an EGFR mutation is an independent unfavorable predictor of PFS (hazard ratio (HR) = 10.47; 95% CI, 4.55–24.07; *p* < 0.001).

### 3.3. Comparisons of the Post-CCRT PFS, and TMDD Based on EGFR Mutation Status

**Figure 3:**
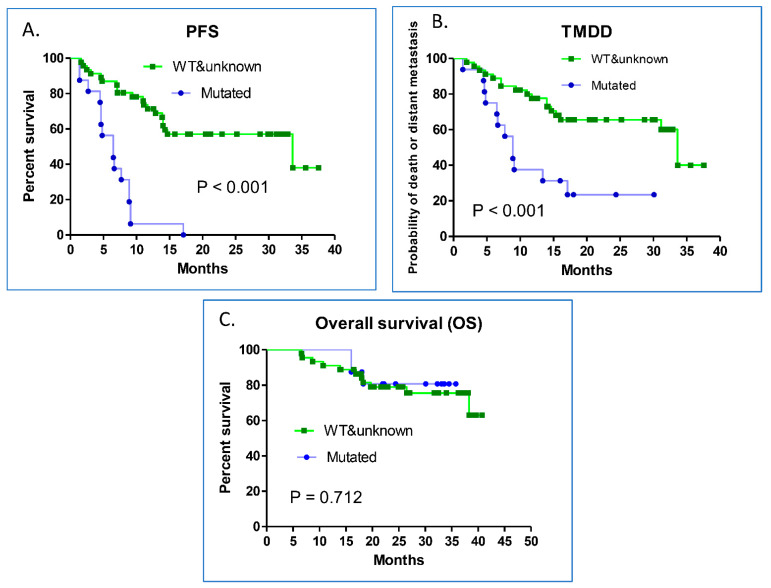
Kaplan–Meier survival curves of PFS, TMDD, and OS. Between the EGFR-mutated, and wild-type and unknown patients, (**A**) the median post-CCRT PFS was 6.50 months for the EGFR-mutated patients and 33.63 months for the EGFR wild-type and unknown patients (HR = 10.47; 95% CI, 4.55–24.07; *p* < 0.001); (**B**) the median post-CCRT TMDD of the EGFR-mutated patients was 8.9 months and 33.63 months for the EGFR wild-type and unknown patients (HR = 5.13; 95% CI, 1.96–13.44; *p* < 0.001); and (**C**) no statistically significant difference was found in the median OS between the EGFR-mutated, and wild-type and unknown groups.

The patients in this study were divided into the EGFR-mutated, or EGFR wild-type and unknown groups for further analysis (Table 3). Histology was the only variable with statistical significance (*p* = 0.026). Among the 16 patients with EGFR mutations, 15 had adenocarcinomas, 1 was not-otherwise-specified NSCLC, and none were squamous cell carcinomas. The median post-CCRT PFS of the EGFR-mutated patients was 6.50 months, which was significantly shorter than that of the EGFR wild-type and unknown patients (33.63 months) (HR = 10.47; 95% CI, 4.55–24.07; *p* < 0.001; Figure 3A). The median post-CCRT TMDD of the EGFR-mutated patients was 8.9 months, which was significantly shorter than that of the EGFR wild-type and unknown patients (33.63 months) (HR = 5.13; 95% CI, 1.96–13.44; *p* < 0.001; Figure 3B). For OS, no statistically significant difference was found between the two patient groups (*p* = 0.712) (Figure 3C). 

Among the 16 EGFR-mutated patients with progressive disease, 4 had local tumor progression and 12 had metastatic disease. In the 12 EGFR-mutated patients with metastatic disease, the most frequent metastatic site was bone (5 patients) followed by brain (4 patients), lungs (4 patients), pleura (3 patients), liver (2 patients), and adrenal gland (1 patient). Among the 12 patients, 5 patients had 2 concurrent metastatic sites and 1 patient had 3 concurrent metastatic sites (pleura, brain, and liver). In the 24 EGFR wild-type and unknown patients, 7 had local tumor progression and 17 had metastatic disease. Regarding the 17 patients with progressive metastasis, the most frequent metastatic site was the brain (5 patients) followed by bone (4 patients), lungs (4 patients), liver (2 patients), adrenal gland (2 patients), pleura (1 patient), and pericardium (1 patient). One patient had three concurrent metastatic sites (brain, liver, and adrenal gland). A total of 29 patients experienced progressive disease with metastasis, and the subsequent systemic therapies following durvalumab in this study are summarized in Table 4.

All 16 EGFR-mutated patients experienced progressive disease after durvalumab therapy in this study, where patients received first- to third-generation EGFR-tyrosine kinase inhibitors (TKIs) as the first subsequent systemic therapy. Two patients received the anti-angiogenesis agent bevacizumab combined with erlotinib therapy. The treatment response to subsequent EGFR-TKIs is shown in Supplementary Table S1. Nine patients had PR, and three patients had SD. The response rate of EGFR-TKIs was 75%, and no patient experienced PD in their initial assessments of response to EGFR-TKIs. The treatment response to EGFR-TKIs was not evaluable in four patients because these patients had small metastases, which were detected using bone scans or FDG-PET scans. All four patients had clinically stable conditions after EGFR-TKIs administration.

### 3.4. Durvalumab-Induced AEs

The durvalumab treatment-induced AEs are shown in Table 5. Among the 61 patients in this study, skin rashes and pruritis (52.5%) were the most frequent AEs, followed by pneumonitis (18%). Regarding the severe grade 3 AEs, three patients experienced pneumonitis and one patient had hepatitis with elevated liver transaminases. Durvalumab therapy was discontinued permanently in the four patients who experienced grade 3 AEs. Two of the three patients who experienced grade 3 pneumonitis were EGFR-mutated. Relapsing grade 2 pneumonitis was recorded in 1 EGFR-mutated patient receiving subsequent afatinib therapy, and the pneumonitis recovered by temporarily interrupting the afatinib and steroid therapy. Pneumonitis did not recur after re-challenging afatinib therapy in the same patient. Most of the durvalumab treatment-related AEs were mild (grades 1 and 2) and manageable. No durvalumab treatment-related mortality occurred in this study.

## 4. Discussion

The analysis of our study provided some insights into post-CCRT unresectable stage III NSCLC patients receiving consolidation durvalumab therapy in real-world practice. Consolidation durvalumab therapy is effective and safe for post-CCRT unresectable NSCLC patients in clinical use. First, our results showed that durvalumab had a 14.0-month post-CCRT PFS and a 16.7-month TMDD. Second, most of the side effects induced by durvalumab therapy in this study were manageable, and only four (6.6%) patients experienced grade 3 AEs and needed permanent discontinuation of durvalumab. Third, in our further analysis, we found that EGFR mutations were an independent factor affecting the efficacy of durvalumab unfavorably. The efficacy of post-CCRT consolidation durvalumab in our study is compatible with the results shown in the PACIFIC trial [16].

The EGFR mutation rate in this study appeared to be higher (26.2%) than that in the PACIFIC trial (6.1% in the durvalumab treatment arm). However, the study subjects recruited in our study were all East Asians, while the PACIFIC trial enrolled patients globally [16]. The frequency of NSCLC EGFR mutations differs according to ethnic groups. The rates of EGFR mutations range from 40% to 55% in East Asians and from 5% to 15% in Caucasians [23,24,25]. Regarding the histological type of the 12 patients with unknown EGFR mutations in our study, 10 were squamous cell carcinomas and 2 were large cell neuroendocrine carcinomas. All 15 squamous cell carcinomas in this study were histologically pure, and none of these were mixed with adenocarcinomas. According to the data from previous studies, the EGFR mutation prevalence in squamous cell carcinomas is very low, ranging from 2% to 7% [26,27]. This indicates that most of the 12 NSCLC patients with unknown EGFR mutations had wild-type EGFR mutations. Previous studies have reported that anti-PD-L1 ICIs do not significantly improve the survival of metastatic NSCLC harboring EGFR mutations when compared with chemotherapy [28,29]. Therefore, most clinical trials with anti-PD-L1 ICIs for the treatment of metastatic NSCLC do not recruit EGFR-mutated patients [14,21]. In a recent study conducted by Aredo et al., they reported that stage III EGFR-mutated NSCLC patients did not benefit from post-CCRT consolidation durvalumab therapy and experienced a high frequency of immune-related adverse events (irAEs) [30]. In our study, the median post-CCRT PFS of consolidation durvalumab in EGFR-mutated patients was 6.5 months, which was significantly lower than that of EGFR wild-type and unknown patients (33.63 months). In addition, we found that EGFR mutations were an independently unfavorable predictive factor for consolidation durvalumab therapy regarding PFS. The study by Aredo et al. recruited only EGFR-mutated NSCLC patients and had no comparison of the consolidation durvalumab between EGFR-mutated and EGFR wild-type patients. In the study by Aredo et al., the number of patients who received consolidation durvalumab was 13, which is less than that in our study (16 EGFR-mutated patients). In the same study, they showed that CCRT followed by EGFR-TKI therapy results in significantly longer post-CCRT PFS than consolidation durvalumab [30]. In our study, all 16 EGFR-mutated patients had progressive disease in durvalumab therapy during follow-up and all patients received EGFR-TKIs as a subsequent therapy. Together, this may explain why no significant difference in OS was found between EGFR-mutated and EGFR wild-type patients, indicating that these patients benefit from EGFR-TKI therapy even after consolidation durvalumab.

irAEs are also concerning in post-CCRT unresectable stage III NSCLC patients receiving consolidation durvalumab. Though most of the irAEs in our study were acceptable, four patients experienced grade 3 AEs and needed permanent discontinuation of durvalumab. Two EGFR-mutated patients in our study experienced grade 3 durvalumab-induced pneumonitis. In the study of Aredo et al., a patient previously treated with durvalumab experienced grade 4 pneumonitis induced by Osimertinib [30]. Interstitial pneumonitis was also recorded in a previous study with the combination of Osimertinib and durvalumab [31]. Though no severe AEs (grades 3 and 4) were induced by subsequent EGFR-TKIs in our study, consolidation durvalumab should be administrated with caution in EGFR-mutated patients. 

Some limitations should be clarified in our study. Besides EGFR mutations, a part of NSCLC is classified as oncogene-addicted NSCLC, which harbors driver mutations, including ALK, ROS1, BRAF, MET, HER2, RET, K-RAS, or NTRK mutations [32,33]. In our study, we did not record patients with driver mutations aside from EGFR mutations. Therefore, further studies may be needed to explore whether other driver mutations have an impact on consolidation durvalumab in post-CCRT unresectable stage III NSCLC. At the very least, our study showed that consolidation durvalumab in post-CCRT unresectable stage III NSCLC is effective and safe for EGFR-wild type patients in real-world clinical practice (33.63-month median PFS and TMDD). Consolidation durvalumab had limited efficacy for post-CCRT unresectable stage III NSCLC with an EGFR mutation according to the results in our study, and a need to look for a better consolidation therapy rather than durvalumab emerges. Third-generation EGFR-TKI Osimertinib has been successfully shown to improve the survival of advanced and resectable early-stage EGFR-mutated NSCLC based on the results of pivotal clinical trials (e.g., the FLAURA and ADAURA trials) [34,35]. Osimertinib used as a consolidation therapy for post-CCRT unresectable stage III NSCLC with EGFR mutations currently under investigation in the LAURA trial [36], and the results of the LAURA trial may support using consolidation osimertinib in the future.

## 5. Conclusions

Consolidation durvalumab is effective and safe for post-CCRT unresectable stage III NSCLC in clinical practice, but EGFR mutations are an unfavorable factor for consolidation durvalumab. Thus, the search for a better consolidation therapeutic strategy for post-CCRT unresectable stage III EGFR-mutated NSCLC is warranted in the future.

## Figures and Tables

**Table 1:** Baseline demographic and treatment information of all patients.

Total	*N* = 61
Gender	
Male	49
Female	12
Age year (median/range)	63 (32–86)
ECOG PS	
0–1	59
2	2
Smoking status	
Non-smoker	18
Former/current smoker	43
Histology	
Adenocarcinoma	42
Squamous cell carcinoma	15
NSCLC *	4
Stage	
IIIA	19
IIIB	35
IIIC	7
EGFR mutation	
Mutated EGFR	16
Wild-type	33
Unknown	12
PD-L1 expression (TPS)	
Positive (≥1%)	27
Negative (<1%)	16
Unknown	18
Chemotherapy regimens Platinum-based doublet with	
Docetaxel	24
Vinorelbine	31
Etoposide	1
Pemetrexed	5
Dose of radiation therapy	
5000–6000 cGy	3
6000–6600 cGy	51
6600–7000 cGy	7
Response to neoadjuvant CCRT	
PR	34
SD	27
Neutrophil-to-lymphocyte ratio (NLR)	
Low (<3.0)	23
High (≥3.0)	38
Timing of first dose of durvalumab administrated post-CCRT, months (median/range)	1.8 (0.2–3.9)
Median follow-up time, months	27.0 (6.7–40.7)

Abbreviations: ECOG PS, Eastern Cooperative Oncology Group performance status; NSCLC, non-small cell lung cancer; EGFR, epidermal growth factor receptor; TPS, tumor proportion score; cGY, centigray; CCRT, concurrent chemoradiation therapy. * Two were large cell neuroendocrine carcinomas, and two were not otherwise specified NSCLCs.

**Table 2:** Cox regression analysis of predictive factors associated with the PFS of durvalumab therapy.

Variables	Patients (*N*)	Median PFS (months)	Univariate Analysis *p*-Value HR (95% CI)	Multivariate Analysis	
				HR (95% CI)	*p*-Value
Age					
<60 years	25	14	0.572		
≥60 years	36	17.6	0.817 (0.406–1.646)		
Gender					
Male	49	13.9	0.535		
Female	12	15.8	0.688 (0.210–2.247)		
Smoking status					
Non-smoker	21	15.8	0.111		
Former/current smoker	40	13.9	2.162 (0.838–5.576)		
Histology					
Adenocarcinoma	42	11.7	0.787		
Non-adenocarcinoma	19	18.1	0.876 (0.334–2.298)		
Stage					
IIIA	19	15.8			
IIIB	35	13.9	0.902		
IIIC	7	7.7	1.041 (0.547–1.981)		
EGFR mutation					
Mutated EGFR	16	6.5	<0.001	10.47 (4.55–24.07)	<0.001
Wild-type and unknown	45	33.63	12.22 (4.296–34.765)		
PD-L1 expression (TPS)					
Positive (≥1%)	27	14			
Negative (<1%)	16	12.9	0.855		
Unknown	18	14.3	0.956 (0.590–1.549)		
NLR					
Low NLR (<3.0)	23	17.1	0.400		
High NLR (≥3.0)	38	12.9	1.375 (0.654–2.891)		
Response to neoadjuvant CCRT					
PR	34	14.7	0.377		
SD	27	14	0.704 (0.324–1.532)		

Abbreviations: PFS, progression-free survival; HR, hazard ratio; EGFR, epidermal growth factor receptor; TPS, tumor proportion score; NLR, neutrophil-to-lymphocyte ratio; CCRT, concurrent chemoradiation therapy; PR, partial response; SD, stable disease.

**Table 3:** Comparison of characteristics between patients with different EGFR mutation statuses.

Variables	Mutated EGFR	Wild Type and Unknown	*p*-Value
Gender			
Male	12	37	
Female	4	8	0.715
Age year (mean ± SD)	60.3 ± 7.1	63.7 ± 12.9	0.587
ECOG PS			
0–1	15	44	
2	1	1	0.459
Smoking status			
Non-smoker	10	33	
Former/current smoker	6	12	0.526
Histology			
Adenocarcinoma	15	27	
Squamous cell carcinoma	0	15	
NSCLC	1	3	0.026
Stage			
IIIA	5	14	
IIIB	9	26	
IIIC	2	5	0.988
PD-L1 expression (TPS)			
Positive (≥1%)	7	20	
Negative (<1%)	5	11	
Unknown	4	14	0.836
Response to neoadjuvant CCRT			
PR	8	26	
SD	8	19	0.77
NLR			
Low NLR (<3.0)	7	16	
High NLR (≥3.0)	9	29	0.565
Timing of the first dose of durvalumab administrated post-CCRT (median, months)	1.6	1.9	0.252

Abbreviations: SD, standard deviation; ECOG PS, Eastern Cooperative Oncology Group performance status; EGFR, epidermal growth factor receptor; NSCLC, non-small cell lung cancer; TPS, tumor proportion score; NLR, neutrophil-to-lymphocyte ratio; CCRT, concurrent chemoradiation therapy.

**Table 4:** Metastatic sites and subsequent systemic therapy following durvalumab in patients with progressive metastasis.

	Mutated EGFR Total Patients *n* = 12	Wild type and Unknown Total Patients *n* = 17
Metastatic sites		
Lung to lung	4	4
Pleura	3	1
Pericardium	0	1
Brain	4	5
Bone	5	4
Liver	2	2
Adrenal gland	1	2
Subsequent systemic therapy following durvalumab	Number of patients (*n*)	Number of patients (*n*)
Afatinib	4	0
Erlotinib	2	1
Gefitinib	1	0
Osimertinib	2	0
Erlotinib + bevacizumab	2	0
AZD3759	1	0
Platinum-based chemotherapy	0	7
Single agent chemotherapy	0	5
Supportive care	0	4

**Table 5:** Treatment-related adverse events (AEs) of durvalumab.

Adverse Events (AEs)	All, *N* = 61 (%)	Grade 1–2, *n* (%)	Grade 3, *n* (%)	Grade 4, *n* (%)
Skin rash/pruritis	32 (52.5%)	32 (52.5%)	0	0
Nausea or anorexia	3 (4.9%)	3 (4.9%)	0	0
Diarrhea	6 (9.8%)	6 (9.8%)	0	0
Amylase or lipase increased	4 (6.6%)	4 (6.6%)	0	0
Liver-transaminases increased	6 (9.8%)	5 (8.2%)	1 (1.6%)	0
Pneumonitis	11 (18%)	8 (13.1%)	3 (4.9%)	0
Headache or dizziness	4 (6.6%)	4 (6.6%)	0	0
Hypothyroidism	4 (6.6%)	4 (6.6%)	0	0
Adrenal insufficiency	6 (9.8%)	6 (9.8%)	0	0

## Data Availability

All data collected or analyzed during this study are included in this published manuscript.

## Supplementary Materials

The following are available online at https://www.mdpi.com/article/10.3390/vaccines9101122/s1, Table S1: Treatment response to subsequent EGFR-TKIs following durvalumab in 16 EGFR-mutated patients after progressive disease.
