# Supplementary material for: Durvalumab as Consolidation Therapy in Post-Concurrent Chemoradiation (CCRT) in Unresectable Stage III Non-Small Cell Lung Cancer Patients: A Multicenter Observational Study
Source: Vaccines (Basel). 2021 Oct 1;9(10):1122. doi: 10.3390/vaccines9101122 (PMC8541274; doi:10.3390/vaccines9101122)
Supplement: Supplementary file 1 [file vaccines-09-01122-s001.zip › vaccines-1376946-supplementary.pdf]

Supplementary Table S1. Treatment response to subsequent EGFR-TKIs following durvalumab in 16 EGFR-mutated patients after progressive disease.

**Total**	**N = 16**
Complete response (CR)	0
Partial response (PR)	9
Stable disease (SD)	3
Progressive disease (PD)	0
Not evaluable (NE)	4
Response rate (RR) %	75
Disease control rate (DCR) %	100
